# A practical approach to supported liquid extraction and measurement of 18 steroids in plasma and serum by targeted liquid chromatography tandem mass spectrometry

**DOI:** 10.1016/j.mex.2024.102728

**Published:** 2024-05-04

**Authors:** Scott G Denham, Joanna P Simpson, Federico Diez, Patricia Lee, Catriona Kyle, Ruth Morgan, Natalie ZM Homer

**Affiliations:** a Mass Spectrometry Core, Edinburgh Clinical Research Facility, Centre for Cardiovascular Sciences, University of Edinburgh, Edinburgh, UK; b Royal (Dick) School of Veterinary Studies, University of Edinburgh, Easter Bush Campus, Midlothian, EH25 9RG, UK; c BHF/University Centre for Cardiovascular Sciences, Queen's Medical Research Institute, 47 Little France Crescent, University of Edinburgh, Edinburgh, EH16 4TJ, UK; d SRUC, Roslin Institute Building, Easter Bush Campus, EH25 9RG, UK

**Keywords:** Targeted, Steroid profiling, Comparative endocrinology, Chromatography, Mass spectrometry, Automation, Supported liquid extraction and LC-MS/MS analysis of multiple steroids in 200 µL plasma

## Abstract

Chromatography combined with mass spectrometry is a gold standard technique for steroid measurement, however the type of sample preparation, the dynamic range and reliability of the calibration curve, the chromatographic separation and mass spectrometry settings ultimately determine the success of the method. The steroid biosynthetic pathway is conserved in higher mammals and literature demonstrates that the concentration ranges of different steroid groups are relatively comparable across species. We sought to develop a robust and reliable multi steroid targeted analysis method for blood that would have wide application across higher mammals. The method was developed following bioanalytical method validation guidelines to standards typically applied to human clinical studies, including isotopically labelled internal standards where at all possible. Here we describe the practical approach to a 96-well supported liquid extraction (SLE) method of extraction from plasma (200 µL) using an Extrahera liquid handling robot (Biotage, Sweden), including quality control samples, followed by a comprehensive separation and targeted LC-MS/MS analysis of 18 steroids in plasma (pregnenolone, progesterone, 17α-hydroxyprogesterone, 11-deoxycorticosterone, corticosterone, 11-dehydrocorticosterone, aldosterone, 11-deoxycortisol, 21-deoxycortisol, cortisol, cortisone, androstenedione, testosterone, 5α-dihydrotestosterone, dehydroepiandrosterone, estrone, 17β-estradiol and estriol).

• SLE in a 96-well format of up to 74 biological plasma samples, enriched with multiple isotopically labelled internal standards, a 12-point aqueous calibration curve, and 6 serum quality controls, designed to monitor long-term performance of the method
• Chromatographic separation of multiple steroids along the gradient, with ammonium fluoride mobile phase additive to improve sensitivity, followed by electrospray ionisation and constant polarity switching
• Aqueous calibration standards that cover physiologically relevant ranges - high nanomolar glucocorticoids, low nanomolar androgens and picomolar ranges for estrogens and steroid intermediates.

## Graphical abstract

**Figure:**
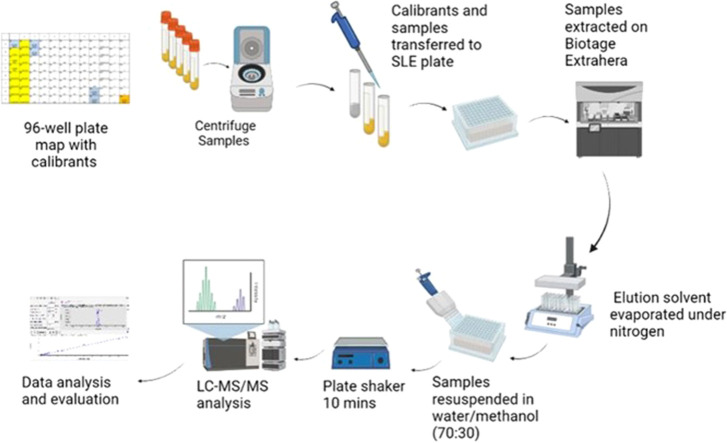

Specifications table

**Table:** 

Subject area:	Biochemistry, Genetics and Molecular Biology
More specific subject area:	*Analytical Chemistry; Endocrinology, Steroid Profiling in blood*
Name of your method:	*Supported Liquid extraction and LC-MS/MS analysis of multiple steroids in 200 µL plasma*
Name and reference of original method:	Ludwig M, Newton C, Pieters A, Homer NZM, Feng Li X, O'Byrne KT & Millar RP. Provocative tests with Kisspeptin-10 and GnRH set the scene for determining social status and environmental impacts on reproductive capacity in male African lions (Panthera leo). *Gen Comp Endocrinol* 2022 **329** 114,127.
Resource availability:	Liquid handling robot, Extrahera (Biotage, Uppsala, Sweden) SPE Dry 96 dual evaporator (Biotage, Uppsala, Sweden) Deepwell Plate Thermoshaker (Grant Scientific) Acquity I-Class UPLC system (Waters, Wilmslow, UK) QTrap 6500+ mass spectrometer (Sciex, Macclesfield, UK) Steroid certified solutions from Sigma-Aldrich/Cerilliant: Cortisol (C-106) 1 mg/mL in methanol (certified) Cortisone (C-130) 100 µg/mL in methanol (Certified) Corticosterone (C-107) 1 mg/mL in methanol (certified) 11-deoxycortisol (D-061) 1 mg/mL in methanol (certified) 21-deoxycortisol (D-062) 100 µg/mL in methanol (certified) 11-deoxycorticosterone (D-105) 100 µg/mL in methanol (certified) Testosterone (T-037) 1 mg/mL in acetonitrile (certified) Androstenedione (A-075) 1 mg/mL in acetonitrile (certified) 5α-dihydrotestosterone (D-073) 1 mg/mL in methanol (certified) Dehydroepiandrosterone (D-063) 1 mg/mL in methanol (certified) Progesterone (P-069) 1 mg/mL in acetonitrile (certified) 17α-hydroxyprogesterone (17OHP) H-085 1 mg/mL in methanol (certified) Pregnenolone (P-104) 100 µg/mL in acetonitrile (certified) 17α-hydroxypregnenolone (H-105) 100 µg/mL in methanol (certified) Aldosterone (A-096) 100 µg/mL in acetonitrile (certified) Estradiol (E-060) 1 mg/mL in acetonitrile (certified) Estrone (E-075) 1 mg/mL in methanol (certified) Estriol (E-074) 1 mg/mL in acetonitrile (certified) ^13^C_3_-Cortisol (C-216) 100 µg/mL in methanol (certified) ^13^C_3_-Cortisone (C-160) 100 µg/mL in methanol (certfied) ^13^C_3_-Corticosterone (C-159) 10 µg/mL in methanol (certified) d5–11-Deoxycortisol (D-078) 100 µg/mL in methanol (certified) d8–21-deoxycortiol (D-076) 100 µg/mL in methanol (certified) d8–17α-hydroxyprogesterone (H-096) 100 µg/mL in methanol (certified) ^13^C_3_-testosterone (T-070) 100 µg/mL in acetonitrile (certified) ^13^C_3_-androstenedione (A-084) 100 µg/mL in acetonitrile (certified) d5-dehydroepiandrosterone (D-064) 100 µg/mL in methanol d9-progesterone (P-070) 100 µg/mL in acetonitrile d8–17α-hydroxyprogesterone (H-096) 100 µg/mL in methanol ^13^C_2_,d2-pregnenolone (P-109) 100 µg/mL in acetonitrile ^13^C_3-_estradiol (E-073) 100 µg/mL in acetonitrile (certified) ^13^C_3-_estrone (E-108) 100 µg/mL in methanol (certified) 11-dehydrocorticosterone (CA3690–000). Powder. From Steraloids ^13^C_3_–5α-dihydrotestosterone (6065) powder from IsoSciences d8-aldosterone (DLM-8438-C) 100 µg/mL in acetonitrile (certified) from Cambridge Isotope laboratories/CK Isotopes ^13^C_3-_estriol (CLM-9147-C) 100 µg/mL in methanol (certified) from Cambridge Isotope laboratores/CK Isotopes Steroid Panel MassCheck Serum 1 level I, II, III (0341, 0342, 0343) from Chromsystems for aldosterone, cortisol, cortisone, corticosterone, 11-deoxycortisol, 21-deoxycortisol Steroid Panel MassCheck Serum 2 level I, II, III (0345, 0346, 0347) from Chromsystems for 11-deoxycorticosterone, testosterone, androstenedione, dihydrotestosterone, dehydroepiandrosterone, progesterone, 17α-hydroxyprogesterone, estradiol
	LC-MS grade Methanol (83638.320) from VWR LC-MS grade water (83645.320) from VWR Ammonium fluoride (338869–25 G) from Sigma-Aldrich HPLC grade Methanol (C-20864320-X) form VWR HPLC grade water (C-10449380-X) from Fisher Scientific HPLC grade Dichloromethane (C-23373320-X) from VWR HPLC grade 2-Propanol (20880.320) from VWR Isolute SLE+ 400 96 well plate (820–0400-P01) from Biotage, Sweden 96 Extrahera 1000 µL pipette tips (414141) from Biotage, Sweden 2 mL deep well 96 well collection plate (186002482) from Waters 96-well plate sealing film (391–1250) from VWR Adhesive Plate Seal (186006336) from Waters Kinetex C18 (150 × 2.1 mm; 2.6 µm) from Phenomenex (00F-4462-AN) Kinetex Krudkatcher ultra 0.5 µm in-line filter (AF0–8497) from Phenomene 2 mL deep well 96-well collection plate (121–5203) from Biotage

## Method details

### Background

Steroid hormones can be classified as corticosteroids (glucocorticoids and mineralocorticoids) and sex hormones (estrogens, progestins and estrogens) [1]. Their synthesis is controlled by the Hypothalamic Pituitary Adrenal axis and the Hypothalamic Pituitary Gonadal axis and they have roles in many physiological and pathophysiological processes. Measuring panels of steroids is important clinically in assessing endocrine function, reproductive health and in cancer [2], [3], [4] and also in veterinary care and welfare in animals [5], [6], [7].

Techniques for steroid measurement include immunoassays, although these are troubled by cross-reactivity and can be impractical for measuring multiple steroids where sample volumes are limited [8]. Alternatively, liquid chromatography tandem mass spectrometry (LC-MS/MS) is able to simultaneously measure multiple steroids with high sensitivity in a single sample. Developing these methods has clear benefits, but their own challenges. Considerations include the definition of the calibration range, mode of sample preparation, separation of the many steroid isomers and isotopologues and confidence in identification of relevant peaks. Fundamentally a steroid panel LC-MS/MS method needs sufficient sensitivity to suit the volume of sample available, appropriate linearity and dynamic range for each steroid, and precision and accuracy to give reliability of the concentrations calculated.

Many steroids are neutral in structure but will take a positive charge in a low pH environment. However, estrogens prefer to take a negative charge and low pH suppresses their deprotonation. To encourage ionizability in the negative mode, without compromising ionisation in positive mode then ammonium fluoride can be used as an additive in the mobile phase [9] enabling a multi-steroid method that includes all major classes of sex steroids (androgens, estrogens and progestins) as well as the corticosteroids.

The technical aim was to analyse these major classes of steroid hormones and their intermediates and to introduce automation to sample preparation in a 96-well format. Automation increases the batch size that can be handled at the bench, to include up to 74 biological samples, while in tandem including quality assurance components so that the method is robust and reliable [10]. Including isotopically labelled internal standards, selecting ^13^C labelled standards wherever available, not only accounts for matrix effects [11], it also supports steroid identification, through tracking the steroids chromatographically [12]. Inclusion of externally provided MassCheck™ quality controls in two panels allows for inter-assay comparability and harmonization between instruments.

The method was developed to cover as many of the key steroids of the biosynthetic pathway (corticosteroids and sex steroids) in higher mammals in blood (plasma and serum) as possible. By ensuring carefully staged design of calibration standards, to cover physiological ranges of the different classes of steroids, the method includes high nanomolar ranges for glucocorticoids, low nanomolar for androgens and picomolar for estrogens across 12 calibration standards. These concentration ranges are broadly preserved across species that have cortisol as the major stress hormone. Through using isotopically labelled internal standards to account for matrix effects and to track chromatographic peaks, the method developed is versatile and has scope for clinical [13,14] as well as veterinary and animal studies and hence comparative endocrinology [15].

### Reagents

All reagents used were of analytical grade or better (*Resource availability section*) cortisol (F), cortisone (E), corticosterone (B), 11-deoxycortisol (S), 21-deoxycortisol (21DF), 11-deoxycorticosterone (11DOC), testosterone (T), androstenedione (A4), 5α-dihydrotestosterone (DHT), dehydroepiandrosterone (DHEA), progesterone (P4), pregnenolone (P5), 17-hydroxypregnenolone (17OHP5), 17-hydroxyprogesterone (17OHP4), aldosterone (Aldo), estrone (E1), estradiol (E2), estriol (E3) and DC Mass Spect Gold Serum (MSG4000) were all purchased from Sigma-Aldrich and Cerilliant. 11-dehydrocorticosterone (A) was purchased from Steraloids, UK.

Internal standards of 2,3,4-(^13^C_3_)-Cortisol (^13^C_3_–F), 2,3,4-(^13^C_3_)-Cortisone (^13^C_3_–E), 2,3,4-(^13^C_3_)-Corticosterone (^13^C_3_–B), 2,2,4,6,6,21,21,21-d8–21-deoxycortisol (d8–21DF), 2,2,4,6,6-d5–11-deoxycortisol (d5–11S), 2,3,4–(^13^C_3_)-Testosterone (^13^C_3_–T), 2,3,4–(^13^C_3_)-Androstenedione (^13^C_3_–A4), 2,2,3,4,4-d5-dehydroepiandrosterone (d5–DHEA), 2,2,4,6,6,17a,21,21,21-d9-Progesterone (d9–P4), 17α-hydroxyprogesterone 2,2,4,6,6,21,21,21-d8–17α-hydroxyprogesterone (d8-17OHP4), 20,21–^13^C_2_, 16,16-d2-pregnenolone (^13^C_2_,d2-P5), 2,3,4–(^13^C_3_)-estradiol (^13^C_3_–E2), 2,3,4–(^13^C_3_)-estrone (^13^C_3_–E1), were purchased from Sigma-Aldrich/Cerilliant. Aldosterone 2,2,4,6,6,17,21,21-(d8–Aldo) and 2,3,4–(^13^C_3_)–estriol (^13^C_3_–E3) were purchased from Cambridge Isotopes Laboratories/CK Isotopes. The internal standard of 2,3,4-(^13^C_3_)-dihydrotestosterone (^13^C_3_-DHT) was purchased from IsoSciences, UK. Stock solutions of all internal standards were prepared at 100 µg/mL in a HPLC grade methanol solution. Working solutions of steroids were prepared in groups, according to the calibration ranges; 0.5 µg/mL (DHT, Aldo, A, E1, E2, E3, S, 11DOC), 1 µg/mL (17OHP4), 2 µg/mL (T, A4), 5 µg/mL (E, DHEA, B, P4), and 100 µg/mL (F). All solutions were stored at −20 °C.

## Preparation of standard solutions, working internal standards and calibration standards for extraction

### Preparation of a 15-steroid standard mixture

Some steroids are not available as certified solutions and must be prepared from powder. Prepare an 11-dehydrocorticosterone stock solution (1 mg/mL) by weighing out approximately 2 mg of 11-dehydrocorticosterone into a glass vial, adding appropriate volume of methanol (HPLC grade) and mixing thoroughly to give a 1 mg/mL solution. A mixture of steroids is then prepared using commercially available solutions and stock solutions of steroids, the concentrations of which are related to the reference ranges of steroids in circulation (Table 2.1).

**Table 2.1:** Preparation of a 15 steroid mix solution of steroids.

Dilution factor and concentration	Volume of solution of standard and starting stock concentration of each steroid
**1:10** (10 µg/mL)	100 µL x 1 mg/mL P4
**1:20** (5 µg/mL)	50 µL x 1 mg/mL B 50 µL x 1 mg/mL DHEA
**1:50** (2 µg/mL)	20 µL x 1 mg/mL T 20 µL x 1 mg/mL A4
**1:100** (1 µg/mL)	10 µL x 1 mg/mL 17αOHP4
**1:200** (0.5 µg/mL)	5 µL x 1 mg/mL A 5 µL x 1 mg/mL S 5 µL x 1 mg/mL DHT 5 µL x 1 mg/mL E2 5 µL x 1 mg/mL E1 5 µL x 1 mg/mL E3
**1:200** (0.5 µg/mL)	50 µL x 100 µg/mL 21-DF 50 µL x 100 µg/mL 11-DOC 50 µL x 100 µg/mL Aldo
Volume of Methanol	570 µL
Final volume	1000 µL

### Preparation of multi-steroid calibration standard solution stocks

The 15-steroid mix prepared in Table 2.1 is combined with stock solutions of F, E, P5 and 17OHP5 to ensure physiologically relevant concentrations of calibration standards (Table 2.2).

**Table 2.2:** Preparation of Standard mixture solutions that are used to prepare the 10 Mix, 1 Mix, 0.1 Mix for preparation of the calibration standards on 96-well plate.

Solution name / Concentration	Volume of 15 steroid mix or certified reference standards	Vol Methanol (µL)	Final vol (µL)
**100 Mix**	100 µL x 1 mg/mL F* 50 µL x 100 µg/mL E 10 µL x 100 µg/mL P5 10 µL x 100 µg/mL 17OHP5 100 µL x [15 Steroid Mix)	730	1000
**10 Mix**	100 µL x **Std Mix 100** above	900	1000
**1 Mix**	100 µL x **Std Mix 10** above	900	1000
**0.1 Mix**	100 µL x **Std Mix 0.1** above	900	1000

*NOTE*: Use certified reference standard solutions for preparation of 100 Mix. Store all dilutions above at −20 °C. Colour code the vials of the dilutions 10 Mix, 1 Mix and 0.1 Mix for ease of calibration curve preparation.

### Preparation of working internal standard solution for one or multiple plates of samples

Either 2 mL or 10 mL of the working internal standard solution (WIS) is prepared for single or multiple plates of samples by combining solutions of isotopically labelled internal standards (See *Reagents* for origin) according to Table 2.3. As with calibration standard solution preparation table above, the preparation of the WIS solution is carried out in two stages, to account for concentration differences between steroids, starting concentrations of certified reference materials, and to ensure the IS concentration for each steroid is as close to the middle of the calibration curve range as possible.

**Table 2.3:** Working internal standard (WIS) solution preparation in 2 mL or 10 mL batches.

Solution name / Concentration	Volume and concentration of each stock solution (µL)	Vol Methanol (µL)	Final vol (µL)
Int Std Mix 1	25 µL x 100 µg/mL ^13^C_3_-E* 100 µL x 10 µg/mL ^13^C_3_-B* 25 µL x 100 µg/mL d9-P* 10 µL x 100 µg/mL d5-S* 10 µL x 100 µg/mL d8–21DF* 10 µL x 100 µg/mL ^13^C_3_-T* 10 µL x 100 µg/mL ^13^C_3_-A4* 10 µL x 100 µg/mL ^13^C_3_-DHT 10 µL x 100 µg/mL d8–17OHP4* 10 µL x 100 µg/mL ^13^C_2_,d2-P5* 10 µL x 100 µg/mL d8-Aldo* 10 µL x 100 µg/mL ^13^C_3_-E2* 10 µL x 100 µg/mL ^13^C_3_-E1* 10 µL x 100 µg/mL ^13^C_3_-E3*	740	1000

Working Int Std (WIS)	**For a single plate** (2 mL total WIS volume) 20 µL x **Int Std Mix 1** 5 µL x 100 µg/mL ^13^C_3_-F 10 µL x 100 µg/mL d5-DHEA	1965	2000
	**For multiple plates** (10 mL total WIS volume): 100 µL x **Int Std Mix 1** 25 µL x 100 µg/mL ^13^C_3_-F 50 µL x 100 µg/mL d5-DHEA	9825	10,000

*NOTE*: Use certified reference materials for preparation of Int Std mix 1 and WIS.

Store all solutions at −20 °C.

### Batch planning and steroid extraction

**Fig. 1:**
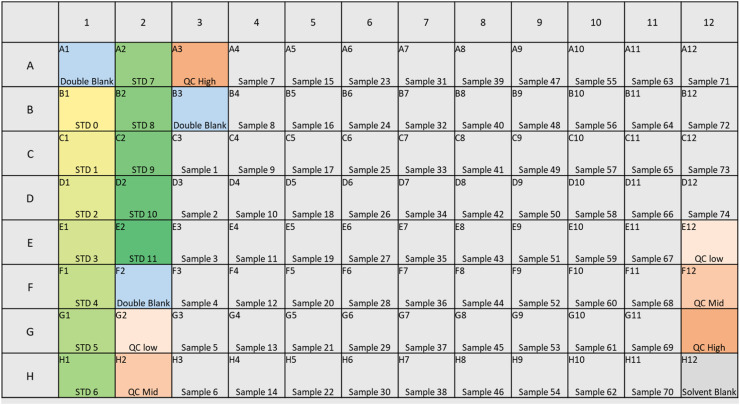
Visual presentation of a plate map plan for the layout of double blanks, solvent blanks, calibration standards, Chromsystems QCs and up to 74 samples steroid extraction for the Extrahera liquid handling robot, prior to LC-MS/MS analysis for steroids.

Design an electronic 96-well plate map for each batch of samples (74 samples or less), to include double blanks, calibration standards, samples and 6 quality controls (Chromsystems Panel 1 and 2, levels I, II and III) according to Fig. 1. Use the template for the electronic plate map (See Supplementary S1) for each batch for experimental reference as well as batch creation on the software that operates the mass spectrometer.

The plate is designed column-wise to match with a column-wise liquid handling pipetting scheme. The electronic plate map is used to create the batch sequence in the mass spectrometry software (e.g., Analyst software), carefully preserving the column-wise injection order, so it is important to save this plate map file to build the analysis sequence later.

Defrost plasma or serum samples and centrifuge if necessary. Take a 96-well 2 mL collection plate Biotage, Uppsala, Sweden), label it with a suitable batch name and into the appropriate well, according to the plate map, transfer 200 µL of water into the blank wells of the 2 mL deep well 96-well collection plate.

Use the calibration standard solution dilution solutions (0.1 Mix, 1 Mix and 10 Mix steroid mixtures) to aliquot the correct volumes for the 12-point calibration standard curve and water into the appropriate wells according to Table 2.4 and following the plate map design for the experiment, column-wise (Fig. 1).

**Table 2.4:** Calibration standard table detailing volume of calibration standard stock solution (Table 2.2) and Working IS solution (Table 2.3) to be added to each standard.

				F	E, B, DHEA, P4	T, A4	17αOH-P4, P5, 17αOH-P5	A, S, 21-DF, 11-DOC, DHT, Aldo, E2, E1, E3
Standard name	Vol WIS (µL)	STD vol (µL)	Vol Water (µL)	Amount (ng)	Amount (ng)	Amount (ng)	Amount (ng)	Amount (ng)
0 STD	20	0	200	0	0	0	0	0
1 STD	20	5 µL x 0.1 Mix	195	0.50	0.025	0.010	0.005	0.0025
2 STD	20	10 µL x 0.1 Mix	190	1.00	0.050	0.020	0.010	0.0050
3 STD	20	25 µL x 0.1 Mix	175	2.50	0.125	0.050	0.025	0.0125
4 STD	20	5 µL x 1 Mix	195	5.00	0.250	0.100	0.050	0.0250
5 STD	20	7.5 µL x 1 Mix	192.5	7.50	0.375	0.150	0.075	0.0375
6 STD	20	10 µL x 1 Mix	190	10.0	0.500	0.200	0.100	0.0500
7 STD	20	20 µL x 1 Mix	180	20.0	1.00	0.400	0.200	0.1000
8 STD	20	25 µL x 1 Mix	175	25.0	1.25	0.500	0.250	0.1250
9 STD	20	5 µL x 10 Mix	195	50.0	2.50	1.000	0.500	0.2500
10 STD	20	9 µL x 10 Mix	191	90.0	4.50	1.800	0.900	0.4500
11 STD	20	10 µL x 10 Mix	190	100	5.00	2.000	1.000	0.5000

Aliquot 200 µL of the six quality controls (QCs) into the appropriate wells according to the plate map of Fig. 1.

Add 200 µL of each plasma sample to the appropriate ‘sample’ well

Add 20 µL of the WIS solution (Table 2.3) to all except for Double Blank and Solvent Blank wells. Seal the plate and shake on a Thermoplate shaker (600 rpm, 5 min). Transfer the sample plate into the liquid handling robot

Programme the liquid handling robot, such as an Extrahera robot, to do the following:

1. Load 200 µL of 0.1 % Formic acid (aq) directly into each well and wait 5 mins before loading the diluted samples onto the SLE400+ plate
2. Load 600 µL of 98:2 Dichloromethane: Isopropanol to each well of the SLE+ 400 plate.
3. Collect eluents under positive pressure into deep well collection plate below SLE.
4. Repeat this procedure twice more to give total eluent volume of 1.8 mL

Transfer the collection plate to a dry down system and reduce solvent to dryness, under a stream of nitrogen at 40 °C

Resuspend in 80 µL of 70:30 Water: Methanol.

Seal the plate and shake for 10 min at 600 rpm on a ThermoShaker to ensure samples are resuspended, prior to LC-MS/MS analysis.

### Instrumentation and analytical conditions for steroid analysis by LC-MS/MS

LC-MS/MS is performed on an I-Class Acquity UPLC (Waters, Wilmslow, UK) interfaced to a QTrap 6500+ (AB Sciex, Macclesfield, UK) mass spectrometer, or equivalent. Set up the chromatography on a uHPLC system like an I-Class Acquity UPLC system (Waters, Wilmslow, UK) and inject 20 µL sample onto a Kinetex C18 (2.1 × 150 mm; 2.6 µm particle size), column fitted with a KrudKatcher Ultra In-Line Filter (0.5 µm porosity) both from Phenomenex, UK. Use a mobile phase system of water with ammonium fluoride (50 µM) (A) and methanol (B) solution with ammonium fluoride (50 µM). Use a flow rate of 0.3 mL/min over 16 min, starting at 50 % B for 2 min, rising to 100 % B over 6 min, held for 2 mins, then returning to 50 % B over 0.1 mins and equilibrating for 4.9 min. The temperature of the column is held at 50 °C. Divert the flow to waste from 0 to 2 mins and 11–16 mins.

**Fig. 2:**
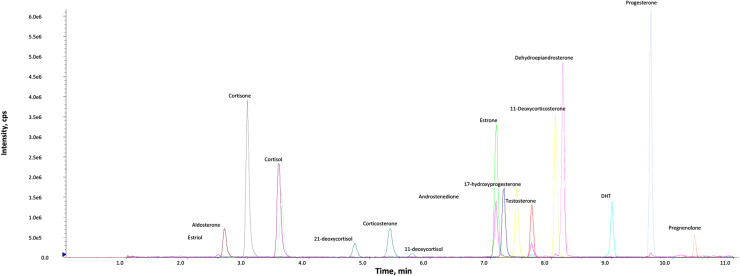
Representative overlaid extracted ion chromatograms of an analytical standard mixture of endogenous steroids in the steroid profiling LC-MS/MS method (internal standards not shown).

Set up the QTrap 6500+ (AB Sciex, Macclesfield, UK) mass spectrometer operated by Analyst 1.7.1 in electrospray ionisation mode with polarity switching using a TurboIonSpray source. Collect data in unit resolution (0.7 *m/z* full width at half maximum). Operate the source at 600 °C with an IonSpray voltage of 5.5 kV/−4.5 kV, a curtain gas of 30 psi, nitrogen nebuliser ion source gas 1 (GS1) and heater ion source gas 2 (GS2) of 40 psi and 60 psi, respectively. Use multiple reaction monitoring (MRM) transitions as detailed in Table 2.5, Table 2.6 with chromatographic retention time for each compound as shown (Fig. 2).

**Table 2.5:** Positive Multiple Reaction Monitoring parameters and retention times for each steroid and isotopically labelled internal standard as analysed on a QTrap 6500+. DP - Declustering Potential; CE - Collision Energy; CXP - Collision Cell Exit Potential. Quantifier (1) and qualifier (2) indicated accordingly. RT according to separation on a Kinetex C18 (150 × 2.1 mm; 2.6 µm), 0.3 mL/min methanol/water system. Assigned internal standard for each steroid indicated in final column.

Steroid	Q1 Mass (*m/z*)	Q3 Mass (*m/z*)	DP (V)	CE (V)	CXP (V)	Time (min)	IS
Corticosterone 1	347.1	121.1	76	29	8	5.31	^13^C_3_-B
Corticosterone 2	347.1	90.9	76	75	12	5.31	^13^C_3_-B
^13^C_3_-corticosterone (^13^C_3_-B)	353.3	125.1	76	29	8	5.16	IS
11-dehydrocorticosterone 1	345.1	121.0	66	31	12	3.55	^13^C_3_-F
11-dehydrocorticosterone 2	345.1	91.2	66	83	40	3.55	^13^C_3_-F
11-Deoxycorticosterone 1	331.2	97.0	86	29	16	7.45	^13^C_3_-T
11-Deoxycorticosterone 2	331.2	109.0	86	31	12	7.45	^13^C_3_-T
17-hydroxyprogesterone 1	331.1	109.0	66	29	12	8.05	d8–17OHP
17-hydroxyprogesterone 2	339.2	100.1	66	31	12	8.05	d8–17OHP
D8–17-OHprogesterone (d8–17OHP4)	339.2	96.9	66	29	12	7.97	IS
Progesterone 1	315.0	97.1	96	23	10	8.90	D9-P4
Progesterone 2	315.0	109.1	96	27	10	8.90	D9-P4
D9-Progesterone (D9-P4)	324.1	100.0	96	23	10	8.90	IS
Pregnenolone 1	317.1	281.1	116	25	18	10.36	^13^C_2_,d2-P5
Pregnenolone 2	317.1	159.0	116	15	18	10.36	^13^C_2_,d2-P5
^13^C_2_,d2-Pregnenolone (^13^C_2_,d2-P5)	321.1	285.2	116	25	18	10.34	IS
Cortisol 1	363.1	121.2	76	31	8	3.46	^13^C_3_-F
Cortisol 2	363.1	91.1	76	83	10	3.46	^13^C_3_-F
^13^C_3_-cortisol (^13^C_3_-F)	366.2	124.0	76	31	8	3.46	IS
11-deoxycortisol 1	347.1	97.0	71	27	12	5.69	D5-S
11-deoxycortisol 2	347.1	109.0	71	33	16	5.69	D5-S
D5–11-deoxycortisol (D5-S)	352.1	100.1	71	27	12	5.62	IS
21-deoxycortisol 1	347.1	311.1	71	23	20	5.20	D8–21DF
21-deoxycortisol 2	347.1	269.0	71	27	14	5.20	D8–21DF
D8–21-deoxycortisol (D8–21DF)	355.2	319.1	71	23	20	5.15	IS
Cortisone 1	361.1	163.1	81	31	26	2.94	^13^C_3_-E
Cortisone 2	361.1	77.1	81	107	10	2.94	^13^C_3_-E
^13^C_3_-cortisone (^13^C_3_-E)	364.2	166.0	81	31	26	2.82	IS
Testosterone 1	289.1	97.0	101	29	12	7.64	^13^C_3_-T
Testosterone 2	289.1	109.2	101	31	6	7.64	^13^C_3_-T
^13^C_3_^−^Testosterone (^13^C_3_-T)	292.1	100.0	101	29	12	7.64	IS
Androstenedione 1	287.1	97.0	61	27	14	6.88	^13^C_3_-A4
Androstenedione 2	287.1	78.9	61	67	10	6.88	^13^C_3_-A4
^13^C_3-_Androstenedione (^13^C_3_-A4)	290.2	100.1	61	27	14	6.88	IS
Dihydrotestosterone 1	291.3	255.2	116	21	30	8.96	^13^C_3_-DHT
Dihydrotestosterone 2	291.3	91.0	116	55	10	8.96	^13^C_3_-DHT
^13^C_3_-Dihydrotestosterone	294.2	258.3	116	21	30	8.96	IS
Dehydroepiandrosterone 1	271.1	235.1	106	17	12	7.96	D5-DHEA
Dehydroepiandrosterone 2	271.1	188.1	106	17	12	7.96	D5-DHEA
D5-Dehydroepiandrosterone	294.1	258.2	21	13	28	7.90	IS

**Table 2.6:** Negative Multiple Reaction Monitoring parameters and retention times for each steroid and isotopically labelled internal standard DP - Declustering Potential; CE - Collision Energy; CXP - Collision Cell Exit Potential. Quantifier (1) and qualifier (2) indicated accordingly. Assigned internal standard for each steroid indicated in final column.

Steroid	Q1 Mass (*m/z*)	Q3 Mass (*m/z*)	DP (V)	CE (V)	CXP (V)	Time (min)	IS
Aldosterone 1	359.1	188.9	−70	−24	−21	2.62	D8-Aldo
Aldosterone 2	359.1	331.0	−70	−22	−35	2.62	D8-Aldo
d8-Aldosterone (D8-Aldo)	367.2	193.9	−70	−24	−21	2.59	IS
Estrone 1	269.1	144.9	−150	−48	−15	7.20	^13^C_3_-E1
Estrone 2	269.1	142.9	−150	−70	−15	7.20	^13^C_3_-E1
^13^C_3_-Estrone (^13^C_3_-E1)	272.1	147.8	−150	−52	−21	7.20	IS
Estradiol 1	271.0	144.9	−110	−52	−21	7.00	^13^C_3_-E2
Estradiol 2	271.0	182.9	−110	−52	−19	7.00	^13^C_3_-E2
^13^C_3_-Estradiol (^13^C_3_-E2)	274.0	147.9	−110	−48	−29	7.00	IS
Estriol 1	287.1	171.0	−110	−52	−21	2.54	^13^C_3_-E3
Estriol 2	287.1	145.0	−110	−52	−19	2.54	^13^C_3_-E3
^13^C_3_-Estriol (^13^C_3_-E3)	290.2	173.9	−110	−48	−29	2.55	IS

### Set up for batch analysis using the experimental plate map plan

Using the experimental plate map in Excel, set up the batch in the mass spectrometer operating software, (e.g., Analyst for Sciex instrumentation), using an LC-MS/MS method as defined in section 2.6. Set the sequence to run in the column-wise injection order defined by the Supplementary S1 excel template. Check that the liquid chromatography column is separating the steroid peaks according to the method retention times (Table 2.5, Table 2.6) and that the sensitivity is sufficiently good, by injecting a system suitability test of the Std 2. Once confirmed that the LC-MS/MS system is performing well begin the LC-MS/MS analysis of the batch, by starting with a solvent blank and moving on to the first well – a double blank and the calibration curve, double blanks, followed by the Panel 1 QCs, the unknowns, the Panel 2 QCs followed by solvent blank. Once the data has been collected it must be evaluated for retention time consistency, linearity, QC accuracy and interfering peaks.

## Data analysis of calibration curve and calculation of steroid levels in samples

### Data evaluation by peak integration of multi-steroid profiling data using MultiQuant 3.0.3

Multi-steroid data collected by the LC-MS/MS method can be evaluated using a purpose-built quantitation method. For example, one that has been designed in MultiQuant 3.0.3 with MRM transitions and retention times (RT) of steroids and isotopically labelled internal standards (IS) steroids defined, according to the mass transitions of the quantitative and qualitative ions and analytical standard retention times of the chromatographic method (Table 2.5, Table 2.6). We follow this protocol (Homer, 2023, Protocols.io) [16]. Any quantitation software package could handle this kind of MRM data in the same fashion. Quantitative/Qualitative ion ratio is calculated by dividing the peak area of the quantitative ion by the qualitative peak area and is based on the ion ratio of the calibration standards. Any steroid in a biological sample that has an ion ratio that exceeds 20 % is excluded from the calculation of steroid quantity and the result is not compiled into the final data set.

Concentration levels of calibration standards and QCs can be input into the quantitation method table as amounts in ng, according to Table 2.4. Blanks (internal standard extracted only), double blanks (extracted solution that contains neither standard nor internal standards) solvent blanks (the resuspension solvent used to resuspend samples in final step prior to LC-MS/MS analysis) and the biological samples are defined as Blanks, Double Blanks, Solvent and Unknowns, respectively.

The quantitation method integrates all peaks at the defined retention times, resulting in a peak area for each IS and steroid detected in each calibration standard point, each QC and each biological sample. Linear regression of the peak area ratio of the steroid to IS against the calibration standard amount is calculated and QCs are checked for compliance. Linear regression is used to calculate the amount of steroid in each biological sample.

Carry out systematic assessment of the integration of each of the peaks of the IS and the steroid analytes in the sample batch:

1. Ensure the automated integration of each isotopically labelled internal standard steroid peak is picking the correct peak by assessing retention times of each internal standard (IS) in the standards and also in the samples. Adjust the RT for the method to capture all peaks where necessary (as columns age the retention time can get shorter in reverse phase chromatography). A consistent change to the RT of an IS in the batch will affect the unlabeled steroid too, so note this for the next step.
2. Check that each peak of IS in each sample of the batch has been integrated. (If you are using MultiQuant you can do this by using the metric plot function of MultiQuant to assess peak area, noting that double blanks and solvent blanks will have a 0 value)
3. Assess the integration of each IS in turn and note any change in RT across the batch
4. For each steroid, repeat the procedure of checking RT and metric plot to ensure integration of each steroid peak is correct. You may need to adjust RT if IS has had a shift in RT.
5. Check that each steroid peak in the batch has been integrated in the calibration standards and QCs. Note the unknowns (i.e., biological samples) will not necessarily have peaks of the steroid of interest but if extraction has been successful, they will have IS peaks.
6. Assess the calibration curve and include within 15 % for linearity, 20 % for LLOQ.
7. Check the quality control (QC) concentration is within 15 % for acceptability.
8. Calculate the concentration of each steroid in each unknown.
9. Copy the Full Summary table of calculated amounts of steroids (ng) and peak areas of all steroids from the processing software (e.g., MultiQuant software) into excel for steroid profile filtering and summary creation.
10. Note that the calculated amount of each steroid, according to Table 2.4 is in ng and must be divided by the volume extracted (µL) and multiplied by 1000 to give the ng/mL concentration once data has been brought together as a summary steroid profile.

### Data sorting in MS Excel of multi-steroid concentration data to create steroid profile

With the full summary data copied from mass spectrometer software (e.g., MultiQuant software) into Microsoft Excel, sort the data according to Steroid Name. Copy each steroid result file in turn into a new tab in the same excel file, rename the summary tab and rename the column of the ‘calculated amount’ with the steroid abbreviation. When all steroids have been sorted into separate tabs, copy the sample name, sample ID, sample comment and the renamed calculated amount column into a new tab that can be called ‘Summary’. Copy the concentrations column for all other steroids in turn into the ‘Summary’ tab. The summary that results will be the calculated amount of steroid in ng. The calculated amount of each steroid, according to Table 2.4 is in ng and must be divided by the volume extracted (µL) and multiplied by 1000 to give the ng/mL concentration. To further convert to nanomolar, the ng/mL amount must be divided by the molecular weight of the steroid and multiplied by 1000. The resulting data, row by row, is the steroid ‘profile’ for each sample analysed using this method.

## Method validation

### Recovery of steroid from supported liquid extraction

Wells were filled with 200 μL gold serum; and either pre-spiked or post-spiked. Six wells were pre-spiked with a low, medium or high concentration of the steroid mixture (Corresponding to the Std 2, Std 6 and Std 10, respectively in Table 2.4) and extracted.. The unspiked extracted wells were ‘post-spiked’ with the same concentration of solutions. Recovery was calculated as the ratio of pre-spiked/post-spiked and multiplied by 100 to express as a percentage (Table 4.1)

**Table 4.1:** Recovery of steroids from gold serum at three concentrations, by automated supported liquid extraction, following dilution with 0.1 % formic acid in water and elution with dichloromethane/isopropanol.

ng/mL	A	11DOC	E3	11S	21DF	Aldo	DHT	E2	E1
**0.0025**	93.9 %	107.8 %	89.1 %	90.9 %	105.5 %	88.8 %	120.8 %	91.9 %	77.7 %
**0.0250**	98.8 %	118.9 %	91.0 %	106.7 %	104.1 %	97.2 %	134.2 %	94.7 %	97.7 %
**2.2500**	94.1 %	97.8 %	90.1 %	93.8 %	93.3 %	99.6 %	123.0 %	88.3 %	88.4 %
**Average**	**95.6 %**	**108.1 %**	**90.1 %**	**97.2 %**	**101.0 %**	**95.2 %**	**126.0 %**	**91.6 %**	**87.9 %**

**ng/mL**	**A4**	**T**		**ng/mL**	**E**	**B**	**P4**	**DHEA**	

**0.10**	137.0 %	106.1 %		**0.25**	89.9 %	95.4 %	96.1 %	92.6 %	
**1.00**	119.8 %	112.2 %		**2.50**	91.3 %	96.0 %	99.5 %	112.8 %	
**9.00**	104.3 %	100.9 %		**22.50**	94.3 %	99.3 %	88.6 %	97.9 %	
**Average**	**120.4 %**	**106.4 %**		**Average**	91.9 %	**96.9 %**	**94.7 %**	**101.1 %**	

**ng/mL**	**P5**	**17OHP4**		**ng/mL**	**F**				

**0.05**	121.7 %	97.7 %		**5**	103.4 %				
**0.50**	96.9 %	101.8 %		**50**	102.3 %				
**4.50**	80.8 %	91.6 %		**450**	95.6 %				
**Average**	**99.8 %**	**97.0 %**		**Average**	**100.4 %**				

### Inter-assay accuracy and precision of method

Calibration curves were plotted as the peak area ratio (PAR) of the peak area of the steroid analyte divided by the peak area of the internal standard vs the steroid concentration (Table 4.2). Calibration lines of best fit were considered acceptable when the regression coefficient (r), was >0.99, with 1/x weighting.

**Table 4.2:** Inter-assay accuracy (%RME) and precision (%RSD) for each steroid following automated supported liquid extraction and LC-MS/MS analysis.

Range	A	11DOC	E3	11S	21DF	Aldo	DHT	E2	E1
LLOQ (ng/mL)	**0.125**	**0.125**	**0.0125**	**0.0625**	**0.1875**	**0.0625**	**1.25**	**0.0625**	**0.25**
Inter-assay precision (%RSD)	17.5 %	7.8 %	19.0 %	18.6 %	17.8 %	13.0 %	8.3 %	8.6 %	15.9 %
Inter-assay accuracy (%RME)	9 %	−2 %	9 %	7.5 %	−3 %	0 %	0 %	3 %	5 %
ULOQ (ng/mL)	**2.5**	**2.5**	**2.5**	**2.5**	**2.5**	**2.5**	**2.5**	**2.5**	**2.5**
Inter-assay precision (%RSD)	5.7 %	4.2 %	3.2 %	2.4 %	6.7 %	10.6 %	8.2 %	3.1 %	7.1 %
Inter-assay accuracy (%RME)	−4.7 %	1.0 %	2.8 %	2.1 %	1.3 %	0.8 %	3.0 %	3.0 %	5.8 %

**Range**	**A4**	**T**	**E**	**B**	**P4**	**DHEA**			

LLOQ (ng/mL)	**0.1**	**0.5**	**0.25**	**0.25**	**0.25**	**1.25**			
Inter-assay precision (%RSD)	10.0 %	10.7 %	2.4 %	15.0 %	11.1 %	18.2 %			
Inter-assay accuracy (%RME)	11 %	−4 %	−1 %	−12 %	3 %	2 %			
ULOQ (ng/mL)	**10**	**10**	**25**	**25**	**5**	**25**			
Inter-assay precision (%RSD)	5.7 %	5.1 %	2.6 %	4.0 %	2.4 %	3.7 %			
Inter-assay accuracy (%RME)	1.1 %	−1.7 %	0.4 %	0.2 %	3.8 %	1.8 %			

**Range**	**P5**	**17OHP4**	**F**						

LLOQ (ng/mL)	**1**	**0.5**	**5**						
Inter-assay precision (%RSD)	14.4 %	12.5 %	10.4 %						
Inter-assay accuracy (%RME)	−10 %	−3 %	2 %						
ULOQ (ng/mL)	**5**	**5**	**500**						
Inter-assay precision (%RSD)	13.6 %	7.6 %	8.0 %						
Inter-assay accuracy (%RME)	−0.3 %	−1.2 %	1.9 %						

### Inter-assay accuracy and precision of serum quality control samples

Six separate batches of analysis were prepared and the inter-assay precision and accuracy were calculated of 14 steroids in the two panels of chromsystem serum controls at low, medium, and high concentrations of the steroids (Table 4.3) (aldosterone, cortisol, cortisone, corticosterone, 11-deoxycortisol, 21-deoxycortisol, 11-deoxycorticosterone, testosterone, androstenedione, 5α-dihydrotestosterone, dehydroepiandrosterone and estradiol). Accuracies (%) of individual measurements were obtained by the ratio of the calculated concentration compared to that of the known concentration of steroid in the low, medium and high chromsystem serum controls across six batches.

**Table 4.3:** Inter-assay accuracy and precision (*n* = 6) of quality control serum at low, medium and high levels, defined in the table,for each QC serum panel following automated supported liquid extraction and LC-MS/MS analysis.

		LOW QC			MEDIUM QC			HIGH QC		
	**Range (ng/mL)**	**Level (ng/mL)**	Acc (%)	RSD (%)	**Level (ng/mL)**	Acc (%)	RSD (%)	**Level (ng/mL)**	Acc (%)	RSD (%)
T	0.05–10	**0.189**	98.6	14.3	**1.51**	96.8	4.5	**8.02**	101.3	9.5
A4	0.05–10	**0.291**	92.4	18.9	**1.14**	104.4	5.6	**9.45**	104.3	9.9
ALDO	0.0125–5	**0.098**	99.8	16.3	**0.223**	110.7	15.8	**0.932**	107.8	5.3
F	2.5–500	**24.90**	105.0	14.8	**59.80**	107.2	19.1	**171.0**	110.9	14.0
E	0.125–25	**2.010**	108.8	16.3	**12.00**	109.8	14.9	**29.00**	106.7	11.0
B	0.125–25	**0.868**	101.2	7.9	**4.360**	102.7	18.6	**30.10**	110.3	13.0
11DOC	0.0125–2.5	**0.076**	100.4	9.3	**0.197**	94.2	11.7	**0.984**	101.1	14.7
S	0.0125–2.5	**0.320**	98.5	16.7	**1.500**	92.7	13.1	**9.850**	108.4	7.2
P4	0.025–5	**0.281**	86.2	16.9	**3.080**	94.0	6.9	**15.30**	101.0	9.0
17OHP4	0.025–5	**0.296**	112.0	11.3	**1.470**	109.9	11.6	**8.860**	114.7	13.8
E2	0.0125–2.5	**0.081**	94.9	7.1	**0.414**	104.1	6.3	**2.610**	102.9	6.3
21DF	0.0125–2.5	**0.090**	107.8	11,4	**0.380**	113,9	14,8	**2.290**	111.2	9.8
DHT	0.0125–2.5	**0.080**	107.6	0.2	**0.374**	96.8	10.9	**1.130**	112.8	5.5
DHEA	0.125–25	**1.930**	108.0	5.5	**11.70**	106.8	11.0	**18.20**	107.5	9.9

The inter-assay precision was calculated as the relative standard deviation (%RSD) between the calculated concentrations of each chromsystem panel level of each of the batches. The method was demonstrated to be accurate (80–120 %), and precise where the inter-assay precision was below 20 %.

## Concluding remarks

The current study describes the simultaneous extraction and successful measurement of 18 steroids from plasma using supported liquid extraction followed by analysis by LC-MS/MS and careful data evaluation. 17α-hydroxypregnenolone is included in the method but rarely detected in plasma. The method was validated following European Medicines Agency guidelines. This novel method uses an aqueous calibration curve and has been applied to different species to profile steroids in plasma including human [13,14], porcine, equine, ovine and feline samples [15].

## Ethics statements

Ethical consent was obtained from NHS Lothian Local Research Ethics Committee for all human samples assessed using this method. Animal samples assessed in this multi steroid methodology in plasma were collected using ARRIVE guidelines and were carried out in accordance with the U.K. Animals (Scientific Procedures) Act, 1986 and associated guidelines; EU Directive 2010/63/EU for animal experiments.

## CRediT author statement

**Scott Denham**: Methodology, Investigation, Validation, Writing - review and editing. **Joanna Simpson**: Data Evaluation, Investigation, Writing – review and editing. **Federico Diez**: Investigation, Validation, Writing- review and editing **Patricia Lee:** Laboratory Work, Sample Management, Writing – review, **Catriona Kyle** Conceptualisation, Method application, Writing - review: **Ruth Morgan**: Method application, Investigation, Writing – review, **Natalie Homer:** Conceptualization, Methodology, Formal analysis, Data curation, Writing – original draft, Writing – review & editing.

## Declaration of competing interest

The authors declare that they have no known competing financial interests or personal relationships that could have appeared to influence the work reported in this paper.

## Data Availability

Data will be made available on request.

## Appendix Supplementary materials

Image, application 1
